# Segmenting electroencephalography wires reduces radiofrequency shielding artifacts in simultaneous electroencephalography and functional magnetic resonance imaging at 7 T

**DOI:** 10.1002/mrm.29298

**Published:** 2022-05-16

**Authors:** Thanh Phong Lê, Rolf Gruetter, João Jorge, Özlem Ipek

**Affiliations:** ^1^ Laboratory of Functional and Metabolic Imaging École polytechnique fédérale de Lausanne (EPFL) Lausanne Switzerland; ^2^ Geneva School of Health Sciences HES‐SO University of Applied Sciences and Arts Western Switzerland Geneva Switzerland; ^3^ CSEM ‐ Swiss Center for Electronics and Microtechnology Neuchâtel Switzerland; ^4^ CIBM Center for Biomedical Imaging ‐ Animal Imaging and Technology École polytechnique fédérale de Lausanne (EPFL) Lausanne Switzerland; ^5^ School of Biomedical Engineering and Imaging Sciences King's College London London UK

**Keywords:** 7T, EEG cap, EEG‐fMRI, electromagnetic simulations, shielding artifacts, ultra‐high field

## Abstract

**Purpose:**

Simultaneous scalp electroencephalography and functional magnetic resonance imaging (EEG‐fMRI) enable noninvasive assessment of brain function with high spatial and temporal resolution. However, at ultra‐high field, the data quality of both modalities is degraded by mutual interactions. Here, we thoroughly investigated the radiofrequency (RF) shielding artifact of a state‐of‐the‐art EEG‐fMRI setup, at 7 T, and design a practical solution to limit this issue.

**Methods:**

Electromagnetic field simulations and MR measurements assessed the shielding effect of the EEG setup, more specifically the EEG wiring. The effectiveness of segmenting the wiring with resistors to reduce the transmit field disruption was evaluated on a wire‐only EEG model and a simulation model of the EEG cap.

**Results:**

The EEG wiring was found to exert a dominant effect on the disruption of the transmit field, whose intensity varied periodically as a function of the wire length. Breaking the electrical continuity of the EEG wires into segments shorter than one quarter RF wavelength in air (25 cm at 7 T) reduced significantly the RF shielding artifacts. Simulations of the EEG cap with segmented wires indicated similar improvements for a moderate increase of the power deposition.

**Conclusion:**

We demonstrated that segmenting the EEG wiring into shorter lengths using commercially available nonmagnetic resistors is effective at reducing RF shielding artifacts in simultaneous EEG‐fMRI. This prevents the formation of RF‐induced standing waves, without substantial specific absorption rate (SAR) penalties, and thereby enables benefiting from the functional sensitivity boosts achievable at ultra‐high field.

## 1 INTRODUCTION

Functional magnetic resonance imaging (fMRI), usually based on the blood oxygenation level‐dependent contrast,^1^ and scalp electroencephalography (EEG) are two noninvasive methods to assess brain function. Combining the intrinsic high spatial resolution of fMRI to the high temporal resolution of EEG in simultaneous EEG‐fMRI acquisitions enables characterizing spontaneous aspects of cerebral activity with high accuracy.^2^, ^3^ This is of particular interest in epileptology,^4^ where this multimodal technique was shown to be efficient at mapping epileptic networks in various epilepsy syndromes,^5^ and of clinical utility in the localization of the epileptic focus prior to surgery.^6^, ^7^

In an EEG‐fMRI setup, the radio frequency (RF) electromagnetic (EM) field is typically shielded by the dense conductive EEG components surrounding the imaging subject.^8^, ^9^, ^10^ Although high magnetic field strengths are beneficial to the fMRI sensitivity, they also result in increased mutual artifacts between both modalities leading to a degradation of their respective data quality.^8^, ^11^ The disruption of the EM field distribution may, furthermore, lead to local tissue heating and therefore represent a safety challenge.^12^, ^13^ At ultra‐high field, several EEG‐fMRI setups were found to be affected by strong attenuation of the transmit field ($B_{1}^{+}\;$), as well as local signal dropouts, although no safety concerns were raised.^8^, ^14^, ^15^

Elongated conductors, such as the EEG wires, can behave as antennas, couple to the electric component of RF EM waves,^16^ and disrupt the RF signals.^9^ Furthermore, if resonant conditions are achieved, the resulting current and voltage standing wave patterns could lead to RF heating and injuries.^17^, ^18^ Reducing the coupling between the EEG cap and MRI coil would reduce MR signal losses and increase the MR sensitivity. To limit currents induced on the EEG leads, previous studies have proposed to increase their overall resistance, notably by using carbon fiber wires^12^ (wire resistance of 160±30 Ω) or by printing the EEG cap with resistive ink^19^, ^20^ (wire resistance of 19.0±4.8 kΩ and 910±210 Ω, respectively). Other approaches such as resistivity tapered stripline^21^ could provide a high impedance at RF frequencies, but a low impedance at lower frequencies to avoid affecting the EEG data quality. Thanks to their very high impedance, printed EEG caps provided better MR image quality, with negligible RF shielding, compared to those made out of copper and even carbon fiber wires.^19^, ^20^ However, their applicability is rather limited, since they are not commercially available to our knowledge.

A systematic investigation of EEG‐induced RF shielding artifacts could allow a better understanding of their underlying mechanisms, and help designing solutions to mitigate their impact.

The present study aims at clarifying the properties of EEG‐induced RF shielding artifacts by using extensive EM field simulations of the EEG‐fMRI setup and real MR measurements. A high‐resolution computational model was then taken apart to systematically investigate the artifacts induced by individual EEG components, with a focus on the EEG wiring. After showing that the latter was the main cause of $B_{1}^{+}\;$shielding, we demonstrate both experimentally and numerically the potential of wire segmentation as a practical solution to reduce RF shielding. Finally, EM simulations on a high‐resolution model of the realistic EEG cap with optimized wiring demonstrated a significant reduction of the RF shielding without substantial specific absorption rate (SAR) penalties.

## 2 METHODS

### 2.1 MR measurements

All MR measurements were performed on an actively shielded Magnetom 7T head‐only scanner (Siemens) with a  68‐cm diameter, ultra‐short bore length (Magnex Scientific), AC84 head gradient set and an custom‐made open‐ended 8‐loop head coil (Rapid Biomedical), interfaced to a single transmit and eight receive channels, as previously described.^14^

$B_{1}^{+}\;$maps were acquired using the SA2RAGE sequence^22^ ($\alpha_{1}/\alpha_{2}=4^{\circ }/11^{\circ }$), with $2.0\times 2.5\times 2.0\;{\text{ mm}}^{3}$ and $2\times 2\times 2\;{\text{ mm}}^{3}$ spatial resolution in the human volunteer and an agar‐gel phantom, respectively.

### 2.2 EM field simulations

EM field simulations were performed using Sim4Life V3.2.4 (Zurich Med Tech) using the finite‐difference time‐domain method with SPEAG CUDA libraries.

Simulations were run for 100 periods or until steady‐state conditions were reached (‐50 dB convergence level). Using a standard desktop computer with two graphics accelerators (Intel Core i7‐3820, 32GB RAM, 2×Nvidia GTX 1080Ti), the simulation time typically ranges from 8 to 12 h.

All $B_{1}^{+}\;$, E , and ${\text{SAR}}_{\text{10g}}$ maps were normalized to 1 W total input power and exported to Matlab R2018b (MathWorks) for postprocessing.

### 2.3 EEG‐fMRI setup

#### 2.3.1 64‐channel EEG cap

**FIGURE 1:**
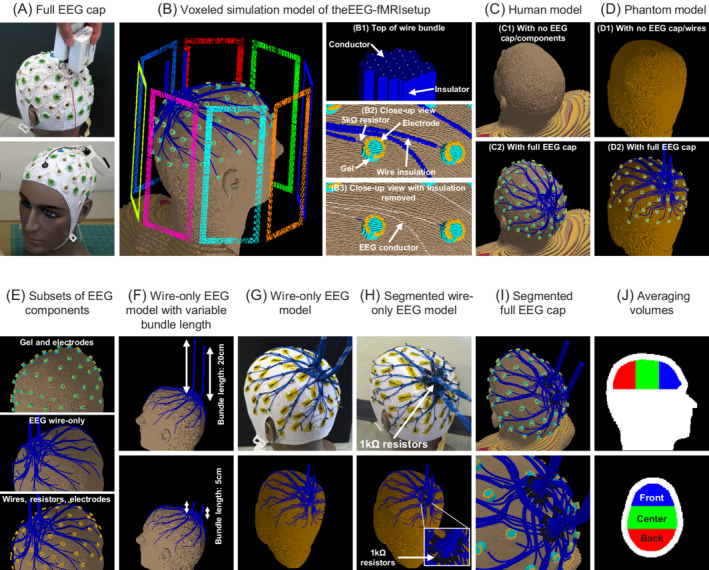
Experimental setups and simulation models. (A) commercial 64‐channel full electroencephalography (EEG) cap. (B) Voxeled simulation model of the EEG‐functional magnetic resonance imaging (fMRI) setup, including the 8‐loop RF coil, 64‐channel EEG cap and realistic human model. (B1) All EEG wires individually converge in two bundles located above the head. (B2) Electrodes are open‐ended and in electrical contact with the skin using a cylinder of conductive gel. A 5‐kΩ current‐limiting resistor connects the wire to the electrode. (B3) Within the wire insulation, each wire is modeled as a line of perfect electric conductor. (C,D) Simulation models without/with the full EEG cap on the human/phantom models. (E) Subsets of the EEG cap. (F) Simulation models with variable wire bundle length. The latter was measured between the scalp and the end of the EEG wires. (G) Wire‐only EEG model for the phantom measurement/simulation. (H) Segmented wire‐only EEG models. Each wire was split at the base of the wire bundle by a 1‐kΩ resistor and insulated with heat‐shrink tubing. (I) Segmented full EEG cap for electromagnetic simulations. (J) Regions of interest used for averaging the transmit field. Volumes were defined with the same thickness on the posterior‐anterior axis.

The EEG setup was composed of a commercial BrainCap MR model (EasyCap, Brain Products), with 65 open‐ring Ag/AgCl electrodes including ground and reference (Figure 1A). Abralyte gel (EasyCap) was used to reduce scalp‐electrode impedance. The EEG wires, designed to be as short as possible (length ranging from 15 to 33 cm), connected each electrode to one of two connector boxes located right above the cap surface. Each EEG lead was made of tinsel copper wire, insulated by a plastic layer and terminated with 5‐kΩ current‐limiting resistors at both ends. Each connector of the EEG cap was connected to a 32‐channel amplifier (BrainAmp MR Plus, Brain Products) using a short  12‐cm bundled ribbon cable as previously described.^14^

#### 2.3.2 Simulation model of the EEG‐fMRI setup

A realistic model of the EEG cap (Figure 1B) was built with 65 electrodes modeled as open rings of perfect‐electric conductors, with 6/8 mm inner/outer diameter, 2 mm thickness and a 2 mm wide slit to reduce eddy currents (Figure 1B2). Within the cylindrical insulation ($\epsilon_{r}=4$), wires were individually modeled as lines of perfect‐electric conductor (Figure 1B3). The distance from the scalp to the wires or electrodes was set to 4.5 mm. Each wire was routed from the electrode to one of the two wire bundles and connectors with a geometry as similar as possible to the physical EEG cap (Figure 1B1). EEG wires were modeled such that there are no direct electrical contacts between two EEG channels. Cylinders mimicking the dielectric gel ($\epsilon_{r}=68$, $\sigma =4.69\;{\mathrm{Sm}}^{-1}$) reduced the scalp‐electrode impedance. A 5‐kΩ current‐limiting resistor with a length of 4  mm was placed at each electrode‐wire connection. This model of the EEG cap described in this paragraph will be referred to as the “Full EEG cap.”

A generic RF coil simulation model was designed with similar physical dimensions to the real RF coil using eight $25.0\times 9.2\;{\mathrm{cm}}^{2}$ rectangular loops of perfect‐electric conductor segmented with three capacitors and voltage sources. This simplified model provides a similar transmit field distribution compared to more detailed models (Supporting information S8 in Data S1). The MR scanner bore was modeled as perfect‐electric conductor to confine the RF fields since this coil does not have an RF shield. The coil provides a circularly polarized excitation at 297.2 MHz, with a decoupling between neighboring elements from −8.3 to −14.3 dB which is similar to the physical coil.^23^, ^24^ A detailed description of the RF coil modeling is in the Supporting information S1 in Data S1.

The imaging object was either the realistic human model Duke 3.0 from the virtual family^25^ or a digitized version of the agar‐gel phantom to match the MR measurements.

The resulting simulation model containing the full EEG cap model, the RF coil, MR scanner bore, and Duke imaging subject will be referred to as the simulation model of the EEG‐fMRI setup (Figure 1B). This setup was discretized into 141.9 MCells with non‐uniform mesh steps from $0.23\times 0.24\times 0.26\;{\text{mm}}^{3}$ to $65\times 65\times 69\;{\text{mm}}^{3}$. The span of the simulation domain on the axial axis is 1.3 m, reaching the middle of the forearm of the imaging subject.

Simulations corresponding to measurements with the agar‐gel phantom used a digital model of the phantom instead of the human model. The latter was digitized based on anatomical images and measured dielectric properties (Supporting information S2 in Data S1). The phantom model with the full EEG cap was discretized into 130.9 MCells with nonuniform mesh steps from $0.25\times 0.24\times 0.24\;{\text{ mm}}^{3}$ to $41\times 53\times 67\;{\text{ mm}}^{3}$, while the phantom model with EEG wiring was discretized into 131.6 MCells with nonuniform mesh steps from $0.18\times 0.21\times 0.21\;{\text{ mm}}^{3}$ to $42\times 53\times 62\;{\text{ mm}}^{3}$.

### 2.4 RF shielding with the full EEG cap

The RF shielding effect of the EEG cap was assessed by comparing the $B_{1}^{+}\;$field amplitude distribution with and without the EEG cap in measurements/simulations on an human volunteer/model (Figure 1C) and an agar‐gel phantom (Figure 1D). One human volunteer participated in this study, and provided oral and written informed consent. The study had been previously approved by the institutional review board of the local ethics committee. For the measurement with the agar‐gel phantom, Abralyte gel was spread on the human‐shaped plastic shell of the phantom to achieve electrical contact between EEG electrodes following standard recommended practice from the EEG manufacturer (Brain Products). The electrodes are electrically insulated from the agar‐gel due to the plastic shell. For each imaging subject, both MR measurements with and without EEG were performed in the same session. The phantom simulation model was digitized from the corresponding anatomical images measured without the full EEG cap.

### 2.5 RF shielding with individual EEG components

To systematically assess the RF shielding induced by individual EEG components, $B_{1}^{+}\;$field amplitude maps from simulations with the realistic human model and following subsets of EEG components were compared: gel and electrodes; EEG wire‐only; EEG wires; wires (current‐limiting) resistors and electrodes (Figure 1E). These datasets were compared against simulations without any EEG components (Figure 1C1) and with the full EEG cap (Figure 1C2).

### 2.6 Characterization and improvement of wire‐only EEG models

The dependence of the shielding artifact as a function of the length of the EEG wiring was assessed in EM field simulations with the realistic human model by varying the length of both EEG wire bundles from 5 to 100 cm by steps of 5 cm (Figure 1F).

For the remaining investigations below, the wire bundle length was set back to 9 cm as on the full EEG cap.

To confirm the role of the EEG wiring in RF shielding artifacts observed in EM simulations, a physical wire‐only EEG model was built to reproduce the wiring of the EEG cap (Figure 1G). 64 wires (ø0.4 mm multistranded copper wire, with Ø1.3 mm OD PVC insulation, length from 150 to 330 mm) were sewn on a subtemporal cap for transmit field mapping within the agar‐gel phantom. A similar simulation model with the wire‐only EEG model placed on the agar‐gel phantom was then simulated for comparison (Figure 1G). The phantom simulation model was digitized from the corresponding anatomical MR images measured without the wire‐only EEG model.

To reduce the interaction between the wiring and the transmit field, each of the 64 wires of the wire‐only EEG model was split into two segments shorter than 170  mm by soldering a commercially available nonmagnetic 1‐kΩ resistor (PNM1206E1001B, 1206 size) specifically designed for MRI applications (Figure 1H). These resistors have been previously verified to have excellent MR compatibility, with negligible effects on MRI data.^26^ All resistors were placed close to the base of the wire bundle. The resistance value was chosen based on simulation results yielding sufficient RF artifact reduction, and availability from major commercial suppliers. Heat‐shrinking tubing was added to avoid short circuits. MR measurements were performed with this segmented wire‐only EEG model on a phantom, in the same session as both measurements with and without the wire‐only EEG model described above. The resistors were added to the numerical model for EM simulations (Figure 1H). Results with all wire‐only EEG models were compared to those with no EEG wires (Figure 1D1)

### 2.7 Segmented full EEG cap

1‐kΩ segmentation resistors were added to the full EEG cap model, and simulated with the realistic human model (Figure 1I). The $B_{1}^{+}\;$amplitude and ${\text{SAR}}_{\text{10g}}$ maps were computed, and compared against the original EEG cap model. Additionally, $B_{1}^{+}\;$and E amplitude field maps within the EEG wire insulation were computed.

### 2.8 Region of interest analysis of field alterations

For all measurements and simulations, the average $B_{1}^{+}\;$amplitude served as a metric for regional signal attenuation, while the relative SD (coefficient of variation) of the $B_{1}^{+}\;$amplitude informed on increases in regional transmit field inhomogeneity. These metrics were computed within three regions of interest (ROI) of the imaging subject, as shown on Figure 1J, as well as a fourth region which is the union of the three above‐mentioned regions. The latter were defined in the realistic human model and divided the brain in three volumes of equal length in the anterior‐posterior direction to distinguish between volumes covered by a high, medium, or low EEG wire density (back, middle, front of the head respectively). To account for the slightly different size and shape between the imaging subjects, the shape of the simulation model was coregistered to anatomical images of the phantom or human volunteer using FLIRT^27^ and FNIRT^28^ from the FSL library.^29^ Using the registration data, the regions of interest were then transformed into the space of the other imaging subjects.

### 2.9 Uncertainty analysis

An uncertainty analysis of the numerical simulation with the segmented EEG cap was performed similarly to previous works.^30^, ^31^ The sensitivity factor of each parameter was computed by comparing the peak ${\text{SAR}}_{\text{10g}}$ between two simulations differing by only this parameter. In distinct simulations, the conductivity and permittivity of head tissues and dielectric gel as well as the resistance of EEG resistors were changed by 10%, while the position of the imaging subject was shifted by 20  mm in three directions or rotated by 10° pitch and roll. The direction of movement taken into account in the uncertainty analysis was the one providing the largest change in peak ${\text{SAR}}_{\text{10g}}$ . In addition, one simulation extended the wire bundles by 24 mm, corresponding to an average extension of the EEG wiring by 10%.

## 3 RESULTS

### 3.1 RF shielding with the full EEG cap

**FIGURE 2:**
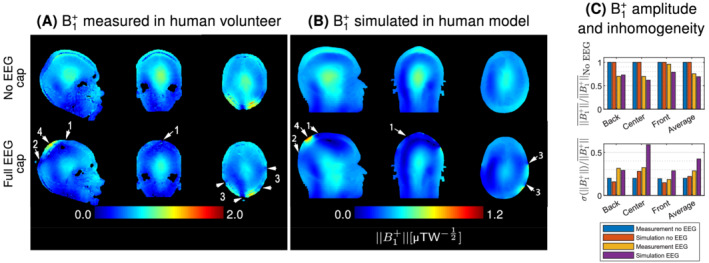
Transmit field maps measured in the human volunteer (A) and simulated in the human model (B) with and without the full electroencephalography (EEG) cap. For MR measurements, the transmit field was expressed as a fraction of the nominal flip angle, while electromagnetic simulation results are normalized to 1‐W input power. For visualization purposes, the scale for simulation results was arbitrarily adjusted such that the same color is applied to the $B_{1}^{+}\;$field value simulated at the center of the human model without EEG compared to the fraction of flip angle measured at the center of the human volunteer without EEG. The lower limit for both scales is zero. An identical colorscale was applied to both no EEG and EEG cases. In overall, a similar transmit field attenuation pattern was observed between measurements and simulations. Shielding artifacts were mostly visible in superior (arrows 1) and posterior (arrows 2) regions of the head, while the frontal region was less affected due to its lower wire density. Furthermore, local dropout regions are observed close to wires and electrodes (arrows 3). A strong $B_{1}^{+}\;$ is depicted close to the wire bundles (arrows 4). Overall, the $B_{1}^{+}\;$amplitude decreased by 24% and 31% with EEG in measurements and simulations respectively, while the inhomogeneity increased by 41% and 93% respectively, mostly affecting the back and center of the subject.

**FIGURE 3:**
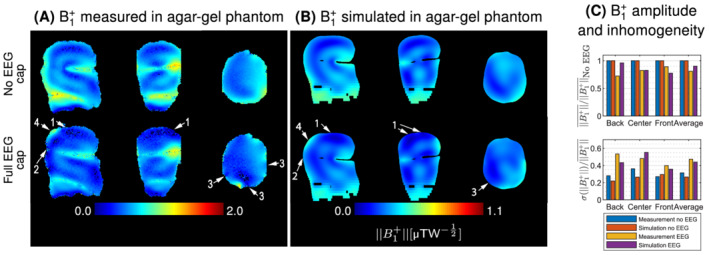
Transmit field maps measured and simulated in the agar‐gel phantom (A) and (B). Measured maps are expressed as a fraction of the nominal flip angle. For visualization purposes, the scale for simulation results was arbitrarily adjusted such that the same color is applied to the $B_{1}^{+}\;$field value simulated at the center of the agar‐gel phantom without electroencephalography (EEG) compared to the fraction of flip angle measured at the center of the agar‐gel phantom without EEG. The color scale remained identical between the results without and with EEG. Radiofrequency shielding artifacts mostly appear in superior (arrows 1) and posterior (arrows 2) regions of the phantom. In addition, EEG components cause localized dropout regions (arrows 3), as well as a strong $B_{1}^{+}\;$ close to the wire bundles (arrows 4). With EEG, the overall $B_{1}^{+}\;$amplitude decreased by 19% and 10% in measurements and simulations respectively, while the inhomogeneity increased by 50% and 65%, respectively.

The RF shielding effect of the EEG cap was investigated by measuring and simulating the transmit field amplitude in a human volunteer/realistic model (Figure 2), as well as in an agar‐gel phantom (Figure 3).

Although the $B_{1}^{+}\;$distribution is different in the agar‐gel phantom compared to the human volunteer/model due to its internal structure with a horizontal air gap separating the different compartments, similar EEG‐induced RF shielding patterns are observed across human/phantom measurements and simulations. All datasets depicted a pattern of strong attenuation and inhomogeneity in superior and posterior regions with the EEG cap (Figures 2 and 3, arrows 1 and 2). Furthermore, local dropout regions were observed close to wires and electrodes (arrows 3), while an intense transmit field was depicted below the bundles where the EEG wiring converges (arrows 4).

The magnitude of the $B_{1}^{+}\;$attenuation in the human volunteer and numerical model was 24% and 31%, respectively, which is higher compared to the 19% and 10% attenuation observed in the phantom measurement and simulation respectively.

Despite the different methods and imaging subjects, all datasets depicted a consistent transmit field disruption pattern, with stronger attenuation and inhomogeneity towards posterior regions of the head, where the wire density is higher.

### 3.2 RF shielding with individual EEG components

**FIGURE 4:**
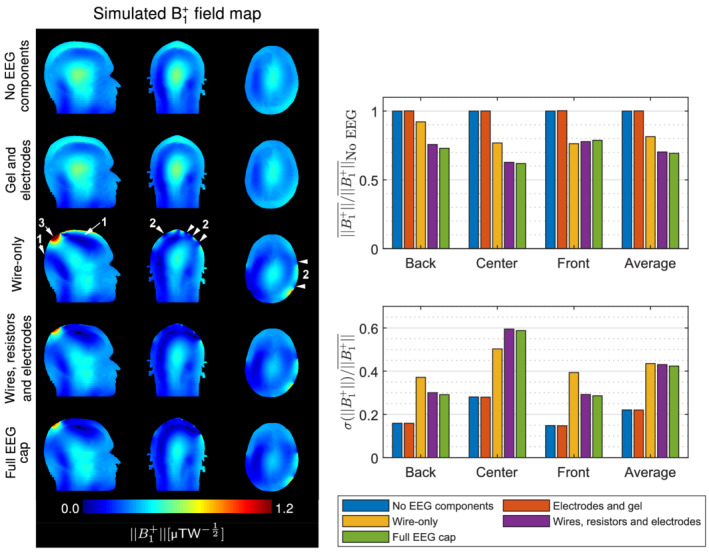
Transmit field maps simulated with different subsets of electroencephalography (EEG) components normalized to 1‐W input power. An identical color scale was applied to all results. The averages were normalized to the values obtained without EEG components. No shielding artifacts were observed when the gel and electrodes were present alone. The EEG wiring alone produced most of radiofrequency shielding artifacts, particularly in posterior and superior regions of the head (arrows 1), local dropout regions near the scalp (arrows 2), and an amplified $B_{1}^{+}\;$at the base of the wire bundles (arrow 3). These artifacts became stronger upon addition of the current‐limiting resistors and electrodes.

EM simulations were performed to systematically investigate the contribution of individual EEG components to RF shielding. No changes in the transmit field were found when EEG electrodes and dielectric gel were placed on the scalp. Strong transmit field shielding was observed in simulations of the wire‐only EEG model, in superior and posterior regions of the head (Figure 4, arrows 1), with local dropout regions near the scalp (arrows 2). Additionally, strong $B_{1}^{+}\;$was observed near the wire bundles (arrow 3). Overall, the wire‐only EEG model attenuated the transmit field by 18.6%, and increased the inhomogeneity by 97% compared to the simulation without EEG components.

Adding the 5‐kΩ current‐limiting resistors and electrodes at the ends of each wire further increased the transmit field attenuation while the inhomogeneity remained similar to the wire‐only model. Finally, adding the dielectric gel to get the full EEG cap model did not result in further differences.

This set of simulations showed that the RF shielding resulted predominantly from the presence of the EEG wiring. Similar transmit field disruption patterns were observed between the wire‐only EEG model and full EEG cap. Therefore the shielding effect can be investigated by considering exclusively the leads and omitting the other EEG components, thereby greatly simplifying the testing models.

### 3.3 Characterization and improvement of wire‐only EEG models

**FIGURE 5:**
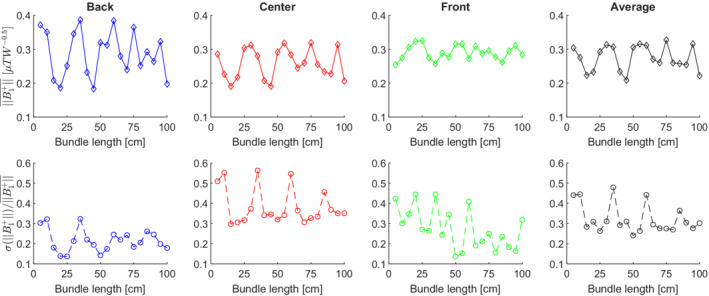
In the wire‐only electroencephalography (EEG) model, the transmit field amplitude and inhomogeneity followed a periodic pattern as a function of the length of the wire bundle. The amplitude of the oscillation was strongest at the back of the head, where the wire density was highest, while no clear pattern was observed at the front with a lower wire density. The periodicity of the oscillations was approximately 25 cm, corresponding to a quarter of the radiofrequency wavelength at 7 T.

The RF shielding artifact as a function of the overall EEG wire length was characterized using EM simulations on the wire‐only EEG model. By varying the wire bundle length, periodic oscillations of the $B_{1}^{+}\;$amplitude and inhomogeneity were observed (Figure 5). A stronger amplitude of the attenuation was observed towards posterior regions of the head where the wire density is higher. The period of the oscillations was approximately 25 cm, corresponding to a quarter of the RF wavelength in air at 7 T. No clear pattern could be observed at the front where the EEG wire density is lower.

**FIGURE 6:**
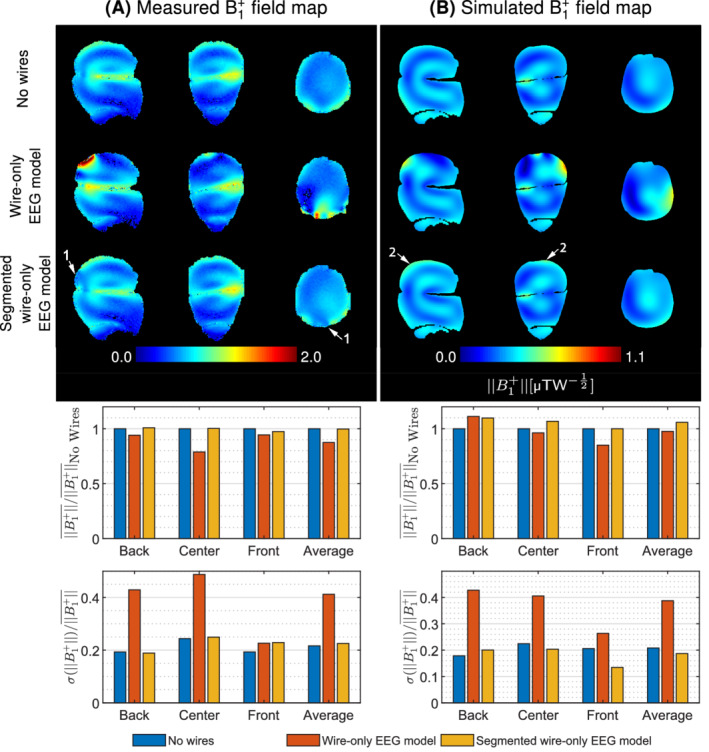
Transmit field maps acquired and simulated in the agar‐gel phantom with the wire‐only and segmented wire‐only electroencephalography (EEG) models. Measured maps are expressed as a fraction of the nominal flip angle. For visualization purposes, the scale for simulation results was arbitrarily adjusted to match the color at the center of the agar‐gel phantom between the measurement without EEG and the simulation without EEG. The color scale remained identical between the results with all three configurations. Strong $B_{1}^{+}\;$attenuation was observed in the presence of the wire‐only EEG model. Most of the shielding artifacts disappeared in the segmented wire‐only model, where each conductor was split by a 1‐kΩ resistor. In MR measurements, only slight shielding effects are remaining in superior and posterior regions (arrows 1), while a slightly stronger $B_{1}^{+}\;$is observed at the upper surface in simulations compared to no wires (arrows 2).

The wire‐only EEG model causes strong RF shielding in both measurement and simulation, although there are substantial differences between both modalities. Nevertheless, the higher wire impedance of the segmented wire‐only EEG model removed most RF shielding artifacts. Slight $B_{1}^{+}\;$attenuation was still observed in posterior regions in measurements (Figure 6A, arrows 1), while simulations depicted a slightly stronger $B_{1}^{+}\;$in superior regions of the head (Figure 6B, arrows 2). The segmented wire‐only EEG model depicted minimal effects on the transmit field, achieving similar $B_{1}^{+}\;$amplitude and homogeneity compared to no wires.

In summary, the periodic pattern of the RF shielding artifacts confirmed the resonant nature of the EEG wiring interaction with respect to the transmit field. MR measurements and EM simulations on the agar‐gel phantom confirmed the effectiveness of segmenting the wire‐only EEG model in suppressing the transmit field disruptions.

### 3.4 Segmented full EEG cap

**FIGURE 7:**
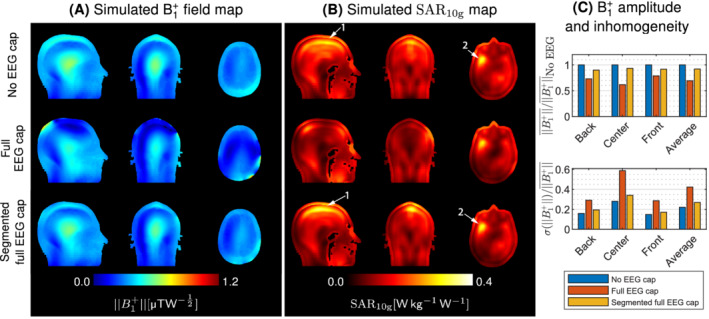
(A) $B_{1}^{+}\;$amplitude and ${\text{SAR}}_{\text{10g}}$ maps simulated in the human model, and normalized to 1‐W input power. With the segmented electroencephalography (EEG) cap, minor shielding artifacts were observed close to the scalp. (B) Most of the power deposition occurred in superior and anterior regions of the head (arrows 1 and 2), and slightly increased in the presence of the segmented EEG cap. (C) The segmented EEG cap model attenuated the $B_{1}^{+}\;$amplitude by only 8% in average compared to 31% with the original EEG cap.

Wire segmentation was applied to the full EEG cap to assess its effectiveness and verify power deposition using EM simulations. Compared to the original EEG cap, the segmented full EEG cap substantially reduced both transmit field attenuation and inhomogeneity, with only slight $B_{1}^{+}\;$disruption remaining in superior and posterior regions of the head (Figure 7A). The transmit field attenuation was reduced from 38.1% to 6.5% in the central regions of interest, and from 30.7% to 8.1% in average by using wire segmentation (Figure 7C).

Alongside the $B_{1}^{+}\;$attenuation, the peak ${\text{SAR}}_{\text{10g}}$ was lower with the full EEG cap compared to no EEG, 0.305 and 0.329 Wkg^‐1^W^‐1^, respectively. Slightly increased power deposition was observed with the segmented full EEG cap, particularly within superior and anterior regions of the head compared to no EEG (Figure 7B, arrows 1 and 2), together with a slight increase of the peak ${\text{SAR}}_{\text{10g}}$ by 4.0% from 0.329 to 0.342 Wkg^−1^W^−1^. The estimated uncertainty on the peak ${\text{SAR}}_{\text{10g}}$ is 12.3% (0.042 Wkg^−1^W^−1^) (Table 1). The highest single uncertainty parameter was the subcutaneous fat conductivity, followed by the subject position along the longitudinal axis.

**TABLE 1:** ${\text{SAR}}_{\text{10g}}$ Uncertainty analysis for the simulation with the segmented EEG cap. Results 1 and 2 denote the peak ${\text{SAR}}_{\text{10g}}$ values computed with the parameter set at Value 1 and 2, respectively. The sensitivity factor was evaluated as the relative change of the result normalized to the relative change of the value,  1 mm or 1° (as indicated). The measurement standard deviation is taken from Neufeld et al.^30^ The total uncertainty is the sum of individual uncertainties, similar to Jeong et al..^31^

			Result 1	Result 2				|Uncertainty|
Parameter	Value 1	Value 2	(${\text{Wkg}}^{-1}$)	(${\text{Wkg}}^{-1}$)	Sensitivity factor		SD	(%)
Skin conductivity (${\mathrm{Sm}}^{-1}$)	0.64	0.70	0.342	0.339	−0.098	(%/%)	0.041	0.63
Subcutaneous fat conductivity (${\mathrm{Sm}}^{-1}$)	0.076	0.084	0.342	0.341	−0.027	(%/%)	0.041	1.44
Muscle conductivity (${\mathrm{Sm}}^{-1}$)	0.77	0.69	0.342	0.342	0.0008	(%/%)	0.041	0.004
Cerebrospinal fluid conductivity (${\mathrm{Sm}}^{-1}$)	2.22	2.45	0.342	0.342	0.003	(%/%)	0.041	0.01
Gray matter conductivity (${\mathrm{Sm}}^{-1}$)	0.69	0.76	0.342	0.337	−0.159	(%/%)	0.041	0.94
White matter conductivity (${\mathrm{Sm}}^{-1}$)	0.41	0.45	0.342	0.338	−0.127	(%/%)	0.041	1.26
Skin permittivity (−)	49.9	54.9	0.342	0.343	0.029	(%/%)	2.8	0.17
Subcutaneous fat permittivity (−)	11.7	12.9	0.342	0.343	0.023	(%/%)	2.8	0.54
Muscle permittivity (−)	58.2	52.4	0.342	0.340	0.052	(%/%)	2.8	0.25
Cerebrospinal fluid permittivity (−)	72.8	65.5	0.342	0.340	0.067	(%/%)	2.8	0.26
Gray matter permittivity (−)	60.1	66.1	0.342	0.339	−0.081	(%/%)	2.8	0.38
White matter permittivity (−)	43.8	48.2	0.342	0.337	−0.141	(%/%)	2.8	0.90
Subject position x (mm) (left‐right)	0	20	0.342	0.392	0.725	(%/mm)	1.15	0.83
Subject position y (mm) (ventral‐caudal)	0	20	0.342	0.345	0.046	(%/mm)	1.15	0.05
Subject position z (mm) (superior‐inferior)	0	20	0.342	0.424	1.189	(%/mm)	1.15	1.37
Pitch (${}^{\circ }$)	0	10	0.342	0.371	0.845	(%/${}^{\circ }$)	1.15	0.97
Roll (${}^{\circ }$)	0	10	0.342	0.368	0.748	(%/${}^{\circ }$)	1.15	0.86
Safety resistors (kΩ)	5	4.5	0.342	0.339	0.098	(%/%)	0.041	0.08
Segmentation resistors (kΩ)	1	0.9	0.342	0.334	0.246	(%/%)	0.041	1.01
Wires extension (mm)	0	24	0.342	0.331	−0.319	(%/mm)	1.15	0.37
Gel conductivity (${\mathrm{Sm}}^{-1}$)	4.69	5.16	0.342	0.342	−0.0014	(%/%)	0.041	0.001
Gel permittivity (‐)	68.0	74.8	0.342	0.342	0.0008	(%/%)	2.8	0.003
Total uncertainties								12.33

**FIGURE 8:**
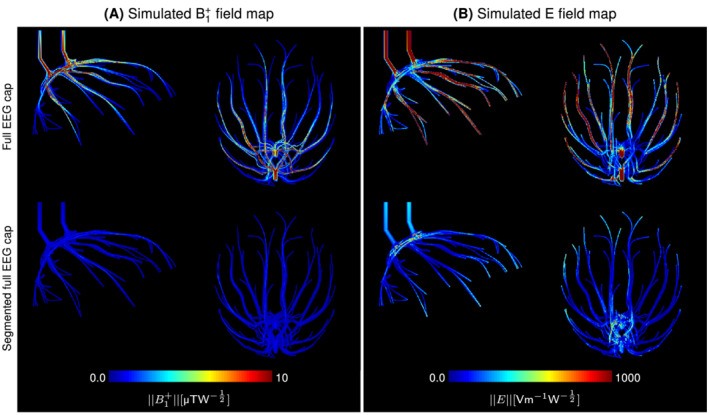
Maximum intensity projection of the transmit field amplitude (A) and the electric field amplitude (B) within the electroencephalography (EEG) wiring insulation, in the simulations with both versions of the EEG cap. With the original EEG cap design, strong transmit and electric field was observed around the longest wires. After segmenting the wires, the transmit field amplitude within the wire insulation decreased from 1.43±2.33 to 0.27±0.27 $\mu {\mathrm{TW}}^{-\frac{1}{2}}$, while the electric field amplitude was reduced from 321±531 to 73±94 ${\mathrm{Vm}}^{-1}W^{-\frac{1}{2}}$.

The amplitude of the transmit and electric fields within the EEG wire insulation were reported in Figure 8. Strong EM fields were observed around the wires in the full EEG cap model, particularly around the longest wires of the EEG cap. Furthermore, strong electric fields were observed within the insulation located between distinct EEG wires, indicating that individual wires were excited to different electric potentials.

Segmentation substantially reduced the current flows along wires, and consequently the EM fields remitted by the EEG wiring. Within the wire insulation, the average transmit field amplitude was reduced by 81%, while the electric field amplitude decreased by 77% in average. The electric field remained slightly higher at the ends of the wire segments and close to the resistors.

## 4 DISCUSSION

In the present work, the causes of RF shielding artifacts in simultaneous EEG‐fMRI were systematically investigated using MR measurements and realistic EM simulations. After analyzing different subsets of EEG components, the transmit field disruption was confirmed to be related to the EEG wiring. Interestingly, the wires were found to resonate with the transmit field while their individual length was substantially shorter than half the RF wavelength. We proposed to segment the EEG wires into shorter sections to suppress the buildup of standing waves, and therefore suppress the transmit field shielding. The effectiveness of this approach was demonstrated using both measurements and simulations on a phantom. Finally, EM simulations on a realistic human model suggested that the redesigned segmented EEG cap provides better MR compatibility, without substantial transmit field shielding, and at the cost of only a slight SAR penalty compared to no EEG.

Numerical simulations of EEG‐fMRI setups are challenging due to the complex geometry of EEG electrodes and dense EEG wiring. Although several studies also employed the finite‐difference time‐domain algorithm, simulations with the finite‐element method (FEM) could potentially better resolve the fine geometric details thanks to the tetrahedral meshing.^32^ Nevertheless, the description of computing hardware from previous works^32^, ^33^ suggests that our equipment was less suited to FEM simulations given the lack of workstations with large RAM amounts (> 32GB) compared to finite‐difference time‐domain for which GPUs were available to accelerate the simulations. To resolve the fine and curved EEG components, the nonuniform meshing parameters were adjusted to provide sufficient separation between EEG wires from different channels and properly resolve the open‐ring electrodes. The fine meshing also contributed to mitigating geometric inaccuracies caused by the hexahedral grid, particularly along the EEG wires. Increasing the geometrical resolution beyond the settings used in this study did not substantially change the $B_{1}^{+}\;$distribution, suggesting convergence of the $B_{1}^{+}\;$with respect to the geometry resolution (Supporting information S3). Furthermore, moving the imaging subject relatively to the coil (Supporting information S4) and using another human model (Supporting information S5) indicated that similar RF shielding patterns could still be observed despite slight geometrical changes.

The first part of this study compared the transmit field disruption induced by the EEG cap across MR measurements and EM simulations in a human/model, and an agar‐gel phantom. Measurement and simulation results depict a reasonable qualitative overall agreement in reproducing the global spatial variations of the transmit field, with minor local disagreements. The latter are mostly located at the edges and air gaps of the imaging subjects. There are quantitative differences between human measurements and simulations, as well as between human and phantom results. This likely arises from dissimilar dielectric structures. Nevertheless, consistent shielding patterns could be reproduced, supporting that further investigations could be performed using the phantom.

Having observed that EM field simulations were in good agreement with experimental observations, the effect of different subsets of the EEG cap was comprehensively studied. The EEG wiring was confirmed to be the main cause of RF shielding, producing a similar attenuation pattern compared to the EEG cap.

Interestingly, the transmit field shielding increased upon connection of the EEG wires to the current‐limiting resistors and scalp electrodes, while these latter were not found to provide substantial shielding by themselves. It could be hypothesized that the current‐limiting resistors enable the RF energy picked up by EEG wires to dissipate into heat instead of being re‐emitted as an EM field, leading to a higher attenuation.

While varying the overall length of wire‐only EEG models in EM field simulations, the transmit field amplitude and homogeneity were found to reach local maxima and minima at intervals of 25 cm, corresponding to a quarter of the free‐space RF wavelength at 7 T. This modulation suggested that standing waves were formed by the transmit field along the wires, and that altering their length could modify their coupling with the transmit coil. Interestingly, the wire length in the original EEG cap configuration (15–33 cm) provided almost the highest transmit field attenuation. Although the wires were expected to resonate at multiples of half the RF wavelength (λ/2=50 cm at  300 MHz),^18^, ^34^ our findings suggested that they rather resonate at multiples of a quarter of the RF wavelength. In this setup, the wires were not uniformly exposed to the electric field, since the wire bundles extend out of the transmit coil, leading to a shortening of the resonant wire length.^35^, ^36^

The length of EEG wire segments could be adjusted to prevent their resonance, for example, by setting them between two resonant lengths.^17^ Unfortunately, the effectiveness of this approach is limited by the dependence of the resonant length on the relative wire position with respect to the transmit coil as well as the variability in length of the EEG wires to cover the whole scalp such that it is difficult to avoid resonance in all conditions, particularly with the shorter wavelengths at 7 T compared to MR at lower field strengths. Instead, keeping them shorter than the fundamental mode identified (λ/4=25 cm) would be a safer approach to avoid standing waves.^37^

Although the current‐limiting resistors placed at both wire ends in the original EEG cap avoid induced currents to leak into the patient or electronics,^16^ they do not prevent buildup of standing waves.^12^ In both measurements and simulations, while the wire‐only EEG model causes strong transmit field disruption, splitting each EEG wire using a 1‐kΩ resistor substantially reduces RF shielding and restores a similar $B_{1}^{+}\;$distribution compared to no wires.

This approach was applied as well to the computational model of a full EEG cap, showing improvement of the transmit field amplitude and homogeneity in EM simulations. The numerical simulations suggested that the modified EEG cap presented a similar power deposition pattern compared to no EEG, with an increase in the local SAR peak of only 4.0% compared to baseline. The uncertainty analysis found high sensitivities for the subcutaneous fat, white and gray matter conductivity, as well as displacements of the subject that would bring EEG wires closer/further from coil elements. The segmented EEG cap was also simulated on the female Ella realistic human model, revealing similar reduction of the RF shielding effects upon addition of the segmentation resistors (Supporting information S5 in Data S1).

Apart from the wire segmentation, and motivated by previous works,^12^, ^19^, ^20^ we note that we also investigated the use of wires with a higher resistivity by measuring and simulating several wire‐only EEG models made out of commercially available materials (linear resistance from 0.14 to 150 Ωm^‐1^, Supporting Information Table S2). Compared to copper wires, none of the more resistive materials were able to simultaneously provide both better transmit field amplitude and homogeneity (Supporting Information Figure S6). It is likely that the resistance of these wires was not high enough with respect to the incoming power to efficiently suppress shielding artifacts. Previous investigations highlighted that carbon fiber wires benefit to the power deposition.^12^ However the MR image quality was still lower compared to no EEG.^20^ The need for a higher lead resistance, hardly achievable using conventional wires, resulted in the development of printed EEG caps.^19^, ^20^ Although they provide good MR performances and compatibility, they are not commercially available to our knowledge.

The methodology of wire segmentation presented here, using commercially available MR‐compatible resistors to suppress electrical currents along the EEG wires, can be applied to existing EEG caps to reduce RF shielding effects. This approach provides more flexibility, since the position, number, and value of the resistors can be adjusted to adapt to a different magnetic field strength or a larger transmit coil. To maximize their efficiency, resistors can be inserted at the expected antinodes of potential standing waves, therefore requiring a smaller overall resistance to achieve the same efficiency as if the resistance was uniformly distributed such as with resistive or printed leads. Since the resistors need to be located close to the patient's head, sufficient protection against mechanical stress and water needs to be provided, for example by encapsulating the resistors and wire ends in a similar manner as at the electrodes.

Segmentation resistors must provide sufficient electrical strength to prevent breakdown during transmission.^38^ At the maximal peak power on our system (combined 8 kW at the RF amplifier outputs, yielding 5.1 kW at the coil plug^39^), the maximal voltage across segmentation resistors reported by the EM simulations is 167 V (Supporting information S7), which is lower than the working voltage of the resistors used in this study (200 V).

The wire segmentation approach is demonstrated here in the case of a single‐channel circularly polarized transmit head‐only volume coil, which is the most common configuration in head studies at 7 T. Nevertheless, power deposition in the subject is highly dependent on how the incident electric field interacts with the wire,^37^ and could therefore be greatly influenced by the wire position and RF coil architecture.^40^, ^41^, ^42^ Although this approach limits resonant conditions and should remain effective regardless of the coil configuration, the power deposition pattern within the patient remains largely dependent on the RF coil and therefore needs to be investigated for each EEG‐fMRI setup. The present work proposes to modify an existing commercial MR‐safe EEG cap that has been used in numerous UHF studies. It is important to point out that the safety of the setup was not fully assessed in the scope of this study. While this does not provide a sufficient safety assessment, it is likely that the existing MR‐safe EEG cap design would remain comparably safe after simply adding resistors to increase the EEG wire impedance. Nevertheless, to ensure RF safety, further assessments such as thermal simulations, temperature measurements, validation of E‐field or SAR distribution should be carried out.^43^

Despite the transmit/receive head‐only coil contributes to a smaller RF exposure volume in comparison to whole‐body transmit coils, the RF fields may extend further out than the coil dimensions.^44^ Therefore, it is necessary to take sufficient precautions to limit the buildup and propagation of RF‐induced currents on remote pieces of wiring in addition to the EEG cap itself. In the present case, the 12‐cm ribbon cables connecting the EEG cap to signal amplifiers are located outside of the coil volume. They are sufficiently short to avoid the antenna effect and terminated at both ends with a high impedance to limit propagation of large RF currents. In addition, the ribbons are located close to the symmetry axis of the coil to minimize the electric field experienced.^35^, ^45^

Finally, it is important to verify whether the adaptation of the EEG cap would affect the EEG data quality. In the unmodified EEG cap, the total EEG wire impedance is already 10 kΩ due to the current‐limiting resistors required at both ends of each EEG wire for patient safety.^16^ Without taking the electrode‐scalp impedance into account, at least a few kΩ for this type of electrode,^46^ adding the 1‐kΩ resistor to segment the EEG wire results in an increase of the total EEG wire impedance by 10%, leading to a 4.9% higher thermal noise. To avoid this slight signal degradation, RF chokes could alternatively be used instead of the resistors to block RF currents without affecting low‐frequency signals.^47^

## 5 CONCLUSION

In this study, causes of transmit field disruption in simultaneous EEG‐fMRI at 7 T were systematically investigated. RF shielding artifacts were found to be mainly caused by standing waves building‐up on the EEG wires. We demonstrated using both MR measurements and EM simulations that using resistors to break the wires into segments shorter than a quarter of the RF wavelength efficiently suppressed most of these artifacts. EM simulations on a realistic human model suggested that the redesigned segmented EEG cap provided better MR compatibility without substantial SAR penalties. To conclude, segmenting the EEG wires is a promising approach to avoid EEG‐induced fMRI data degradation and fully benefit from the functional sensitivity boosts achievable at ultra‐high field.

## Supporting information

S1 S1 RF Coil model
S2 Head‐shaped agar‐gel phantom
S3 Geometry resolution
S4 Position of the imaging object
S5 Different human model
S6 Resistive EEG‐only wire models
S7 Voltage across segmentation resistors
S8 Detailed RF coil models
