# Supplementary material for: Segmenting electroencephalography wires reduces radiofrequency shielding artifacts in simultaneous electroencephalography and functional magnetic resonance imaging at 7 T
Source: Magn Reson Med. 2022 May 16;88(3):1450–64. doi: 10.1002/mrm.29298 (PMC9323442; doi:10.1002/mrm.29298)
Supplement: Supplementary file 1 — S1 S1 RF Coil model S2 Head‐shaped agar‐gel phantom S3 Geometry resolution S4 Position of the imaging object S5 Different human model S6 Resistive EEG‐only wire models S7 Voltage across segmentation resistors S8 Detailed RF coil models [file MRM-88-1450-s001.pdf]

Supporting information for: Segmenting EEG wires reduces RF shielding artifacts in simultaneous EEG-fMRI at 7 T

Thanh Phong LÊ, Rolf GRUETTER, João JORGE, Özlem IPEK

S1 RF Coil model

The RF coil of the EEG-fMRI setup is composed of 8 rectangular loops surrounding the head, similar to the design presented by Weisser et al.[1]. A picture of the physical coil can be found in[3]. This coil is open on both ends to allow passage of the EEG wiring. During transmission, the coil is driven by a single transmit channel, with the power equally divided across the loops by an 8-fold power splitter and phase shifters to match the geometric position of the elements to achieve a circularly polarized transmit field. In transmit mode, the isolation between neighboring elements is about -7 to -8.5dB[1,2]. During reception, the signal from each loop is read by 8 independent receiver channels, and the decoupling between array elements is improved using preamplifier decoupling.

A generic RF coil simulation model with a similar geometry compared to the real coil was built with eight rectangular loops of perfect electric conductors, with an external dimension of $25.0 \times 9.2\text{cm}^2$, and internal size of $23.0 \times 7.2\text{cm}^2$ (Figure S1a). This simplified model provides similar transmit field profiles compared to more detailed models of the RF coil (Supporting information S8). Each loop was segmented at three locations: at the middle of both long edges to insert series capacitors, and at the middle of the short upper edge (opposite to the patient's body) to insert a parallel capacitor as well as the feeding voltage source for RF excitation (Figure S1b). The coil was set in quadrature mode to induce a circularly polarized transmit field, by setting a relative phase of 45° between two adjacent sources (The source $m+1$, $m = 1..7$, has a 45° phase lag compared to source m). Each voltage source applies a harmonic excitation at 297.2 MHz. The scanner bore was modeled as a 680mm ID tube of perfect electric conductor to confine EM fields.

The coil was loaded with the Duke human model (without EEG) to determine optimal capacitor values. Separate multiport simulations were performed for each lumped element and source. With the help of the MATCH tool of Sim4Life, capacitor values were adjusted to match each loop to their respective source output impedance (50 Ω). The optimal values are summarized in table S1a. A set of eight multiport simulations (one for each voltage source) with these values were performed to determine the scattering parameters of the array (Table S1b). The isolation between neighboring elements ranges between -8.3 to -14.3dB.

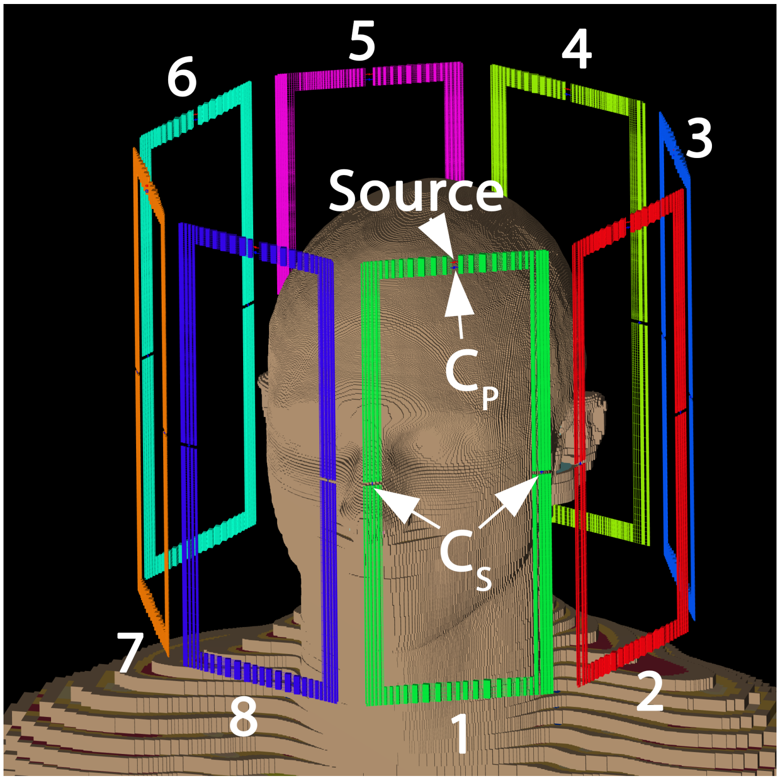

(a)

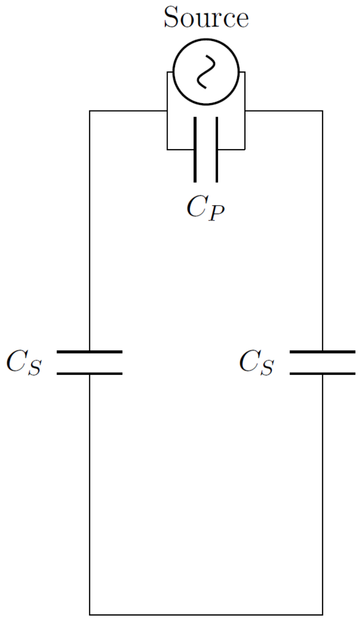

(b)

Figure S1: (a) Voxelized view of the 8-loop RF coil model. Numbers indicate the ordering of the voltage sources of the loops. (b) Schematic drawing of one of the eight loops of the volume coil array, with the position of the source and three capacitors.

Loop	Capacitors	
	C_S [pF]	C_P [pF]
1	0.595	12.2
2	0.589	14.9
3	0.601	13.6
4	0.603	10.3
5	0.603	10.6
6	0.601	14.0
7	0.589	14.9
8	0.594	12.1

(a)

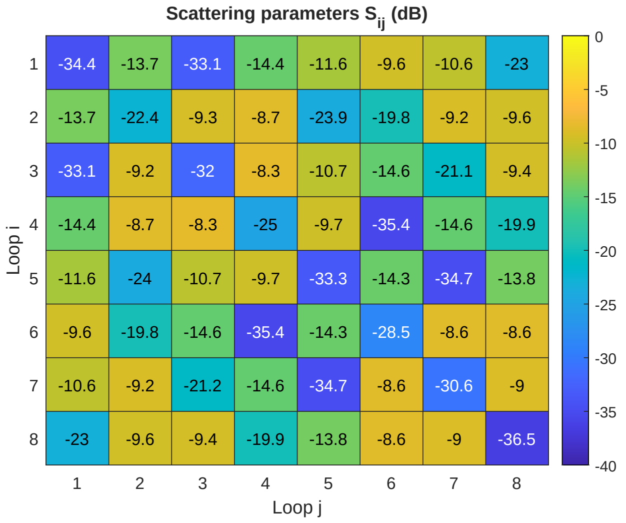

(b)

Table S1: (a) Values of the tuning and matching capacitors of each loop of the volume coil array. (b) Scattering parameters of the simulated 8-loop coil during transmission.

S2 Head-shaped agar-gel phantom

The agar-gel phantom is composed of three distinct parts with the following composition:

- Top part: 3.0 g/l agarose, 9g/l NaCl and 0.13mM Gd-DO3A-butrol in 1850ml H_2O
- Middle and bottom part: 6.46 g/l agarose, 9g/l NaCl and 0.24mM Gd-DO3A-butrol in 2000ml H_2O

Each part was poured into a plastic bag and cast inside a plastic shell in the shape of a human head. The interface between the bags can be seen on anatomical images (Fig.S2). The dielectric properties of the phantom were measured on separate mixtures at 300 MHz and ambient temperature (23 °C) using a dielectric probe (DAK-3.5, SPEAG, Switzerland). For both mixtures, the conductivity was σ=1.68 S m^{-1} and the relative permittivity (dielectric constant) was $\epsilon_r = 76.0$.

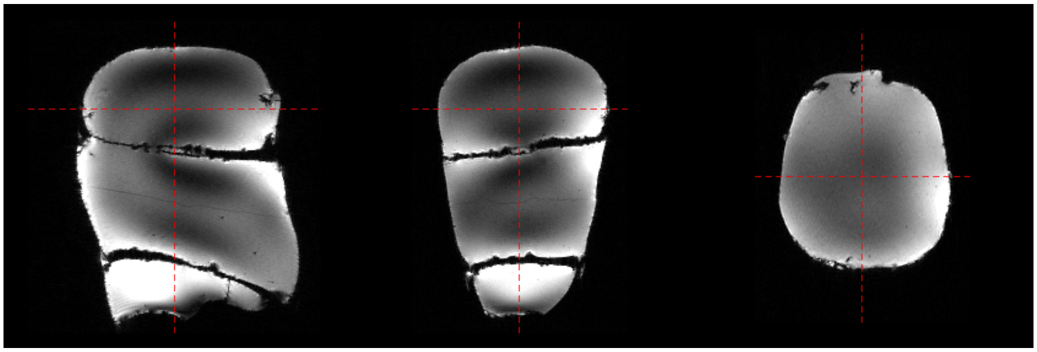

Figure S2: Anatomical slices depicting the internal structure of the agar-gel phantom. The phantom is composed of three distinct parts inside a plastic casing (not visible in the MR images)

Numerical models of the phantom were built to provide EM simulations matching the MR measurements with the EEG cap and EEG wiring on the phantom. Anatomical images were imported into MATLAB and segmented using the Image Segmenter app. The result was then imported into Meshmixer (v3.5, Autodesk, Mill Valley, USA) to be smoothed. A copy of the model was expanded by 6mm to simulate the human-shaped plastic shell. Finally, both models were imported into Sim4life. The electrical properties of the agar-gel were set with the values measured above, while the plastic shell was defined as being polyethylene.

Varying the simulation model of the EEG-fMRI setup

Different variations of the simulation model of the EEG-fMRI setup (including the Duke human model, 8-loop RF coil, full EEG cap and MR scanner bore, as defined in the main manuscript) were built and simulated to verify whether the RF shielding effect could be reproduced across different gridding settings, the relative position of the imaging subject within the coil, and on a different human model.

S3 Geometry resolution

The EEG-fMRI setup with Duke human model was gridded with different geometry resolution of 1.47, 0.85, 0.55 and 0.40mm for the EEG cap wiring (including the insulation), resulting in simulation models with 38, 76, 142 and 290 MCells. Figure S3 depicts the convergence of the $\mathbf{B_1^+}$ field distribution with different gridding settings. While the simulation with 38 MCells yields substantially different $\mathbf{B_1^+}$ amplitude and COV compared to the simulations with finer grids, the results achieved with 142 and 290 MCells are practically identical. Therefore a geometry resolution of 0.55mm for the EEG cap (simulation model with 142 MCells) wiring is sufficiently accurate.

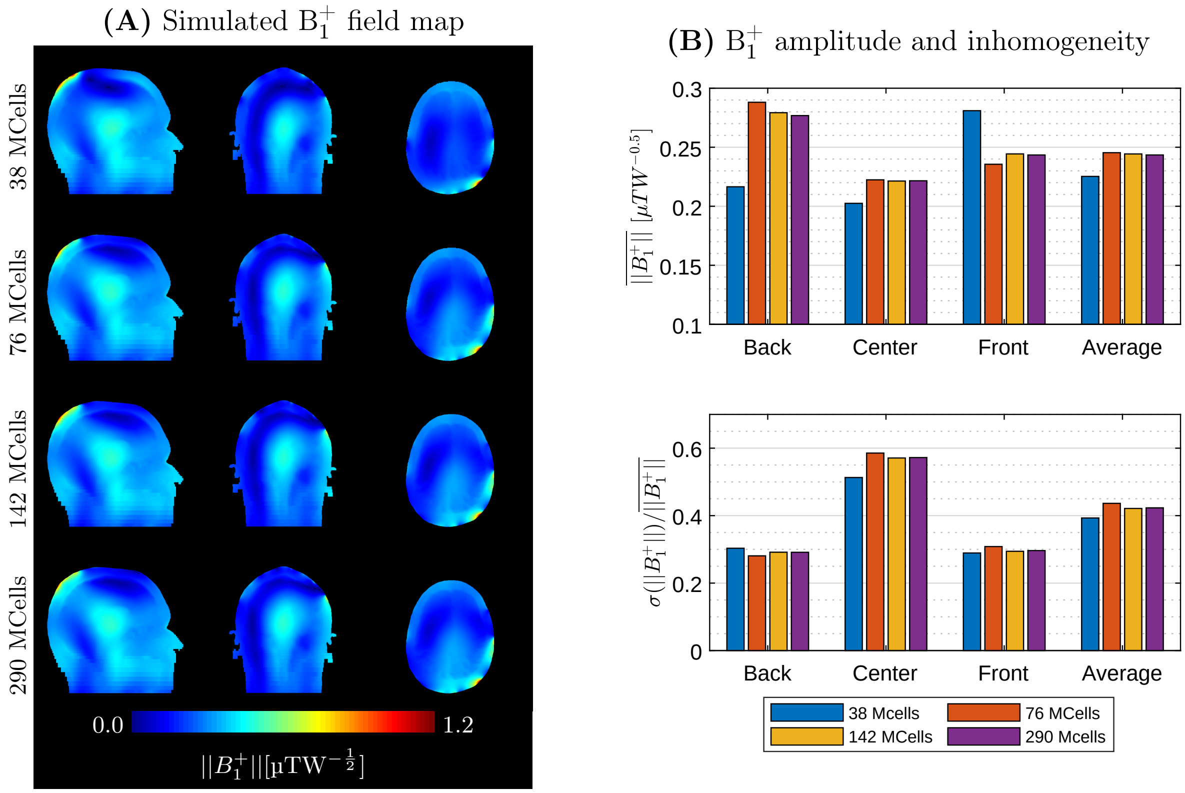

Figure S3: Transmit field distribution with different gridding settings of the EEG-fMRI simulation model.

S4 Position of the imaging object

Both EEG cap and Duke human model were moved relatively to the RF coil. In separate simulations, the imaging subject and EEG cap were translated by $\pm 20\,\mathrm{mm}$ along each direction (craniocaudal, anteroposterior and left-right), and rotated by $\pm 10°$ roll and pitch. The simulation model was gridded for each configuration, with 134.0 to 188.5 MCells (a higher number of voxels was required for the rotated configurations to properly resolve the wire bundles). Each of the ten positions was simulated with and without EEG cap.

Figure S4 depicts the $\mathbf{B_1^+}$ distribution in the different positions. Changes in the $\mathbf{B_1^+}$ distribution are observed in both simulations without and with EEG. In all cases, the RF shielding pattern remains similar to the baseline, with a strong attenuation across the upper part of the head, and strong $\mathbf{B_1^+}$ near the base of the wire bundle, and increased inhomogeneity.

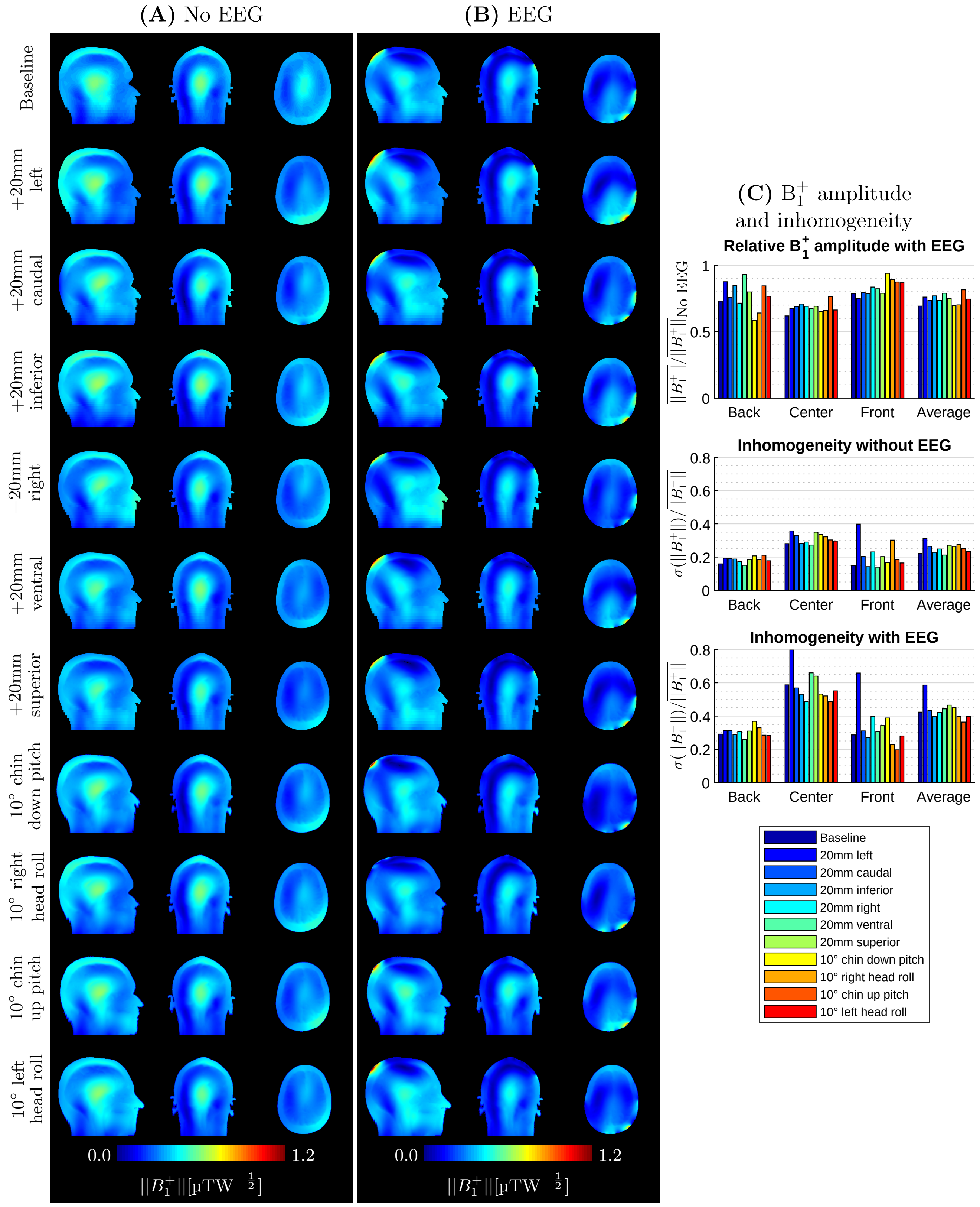

Figure S4: Transmit field amplitude with and without EEG with the imaging subject translated by ±20 mm in all directions, and rotated by ±10° roll and pitch.

S5 Different human model

The EEG-fMRI setup was simulated with the female Ella human model. The EEG wiring and electrodes position were adapted to the smaller and different head shape. The simulation model was discretized into 133.0 MCells with non-uniform mesh steps from $0.27 \times 0.25 \times 0.25\ \mathrm{mm}^3$ to $57 \times 70 \times 70\ \mathrm{mm}^3$.

The transmit field and SAR distribution without EEG, with EEG cap and with the segmented EEG cap are shown in Figure S5. The simulation with EEG depicts a similarly shielding pattern compared to the model with Duke, with a strong attenuation in superior regions of the head, local dropout regions close to the wires and electrodes, and intense transmit field close to the wire bundles. However little attenuation is observed in posterior regions of the head. In overall, the $\mathbf{B_1^+}$ attenuation is attenuated by only 13.7% with Ella model compared to 31% with Duke model.

In the simulation with the segmented EEG cap, the EEG-induced RF shielding is substantially reduced, with only 4.35% $\mathbf{B_1^+}$ amplitude losses compared to no EEG.

A lower power deposition in the upper part of the head was observed with the EEG cap, while the segmented EEG cap caused a higher power deposition close to the scalp. The peak SAR 10g was $0.339\ \mathrm{W\,kg^{-1}\,W^{-1}}$ without EEG, $0.312\ \mathrm{W\,kg^{-1}\,W^{-1}}$ with the EEG cap, and $0.315\ \mathrm{W\,kg^{-1}\,W^{-1}}$ with the segmented EEG cap. The volume with the peak SAR value was situated close to the nasal cavities in all three configurations.

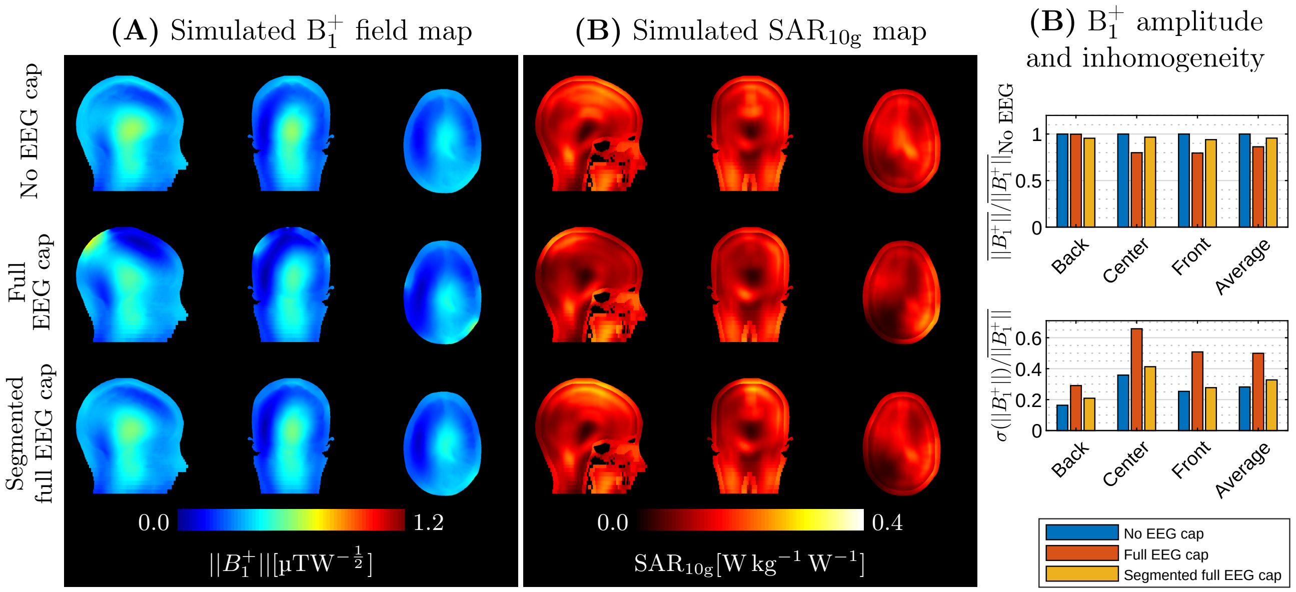

Figure S5: Transmit field and SAR$_{10g}$ distribution within the female Ella human model. The EEG-fMRI setup was simulated without EEG, and with both unsegmented and segmented EEG caps.

S6 Resistive EEG-only wire models

This supporting information provides an assessment of the effect of EEG wire resistivity on RF shielding artifacts, by comparing wire-only EEG models made out of different materials. Their properties are summarized in table S2. Due to the limited availability of certain materials, only the 32 longest wires out of 64 were included, as they were sufficient to cause similar shielding artifacts. We also note there were important differences in wire insulation: the copper and carbon fiber wires had a thick layer of PVC insulation (0.30mm and 0.25mm respectively), while the nichrome and constantan wires were covered by a thin layer of polyimide and enamel respectively to avoid electrical contacts across individual conductors.

Similarly to the method described in the main paper, the wires were sewn to a subtemporal cap placed on the agar-gel phantom, and the transmit field maps were acquired in a 7T MR scanner. Matching EM simulations were performed, in which the 32 wires were set as lossy conductors, and the thick or thin electric insulation layer defined accordingly.

The results are summarized in figure S6.

Material	Conductivity [S/m]	Resistivity [Ω m]	Wire diameter [mm]	Linear resistance [Ω/m]
Copper	5.81×10^{7}	1.72×10^{-8}	0.40	0.14
Nichrome (80Ni/20Cr)	9.17×10^{5}	1.09×10^{-6}	0.50	5.6
Constantan (55Cu/44Ni/1Mn)	2.04×10^{6}	4.90×10^{-7}	0.10	63
Carbon fibers (CPVC4050R, WPI, USA)	3.39×10^{4}	2.95×10^{-5}	0.50	150

Table S2: Electrical properties of the wire-only EEG models. The linear resistance ranged from $0.14\,\Omega\,\mathrm{m}^{-1}$ to $150\,\Omega\,\mathrm{m}^{-1}$

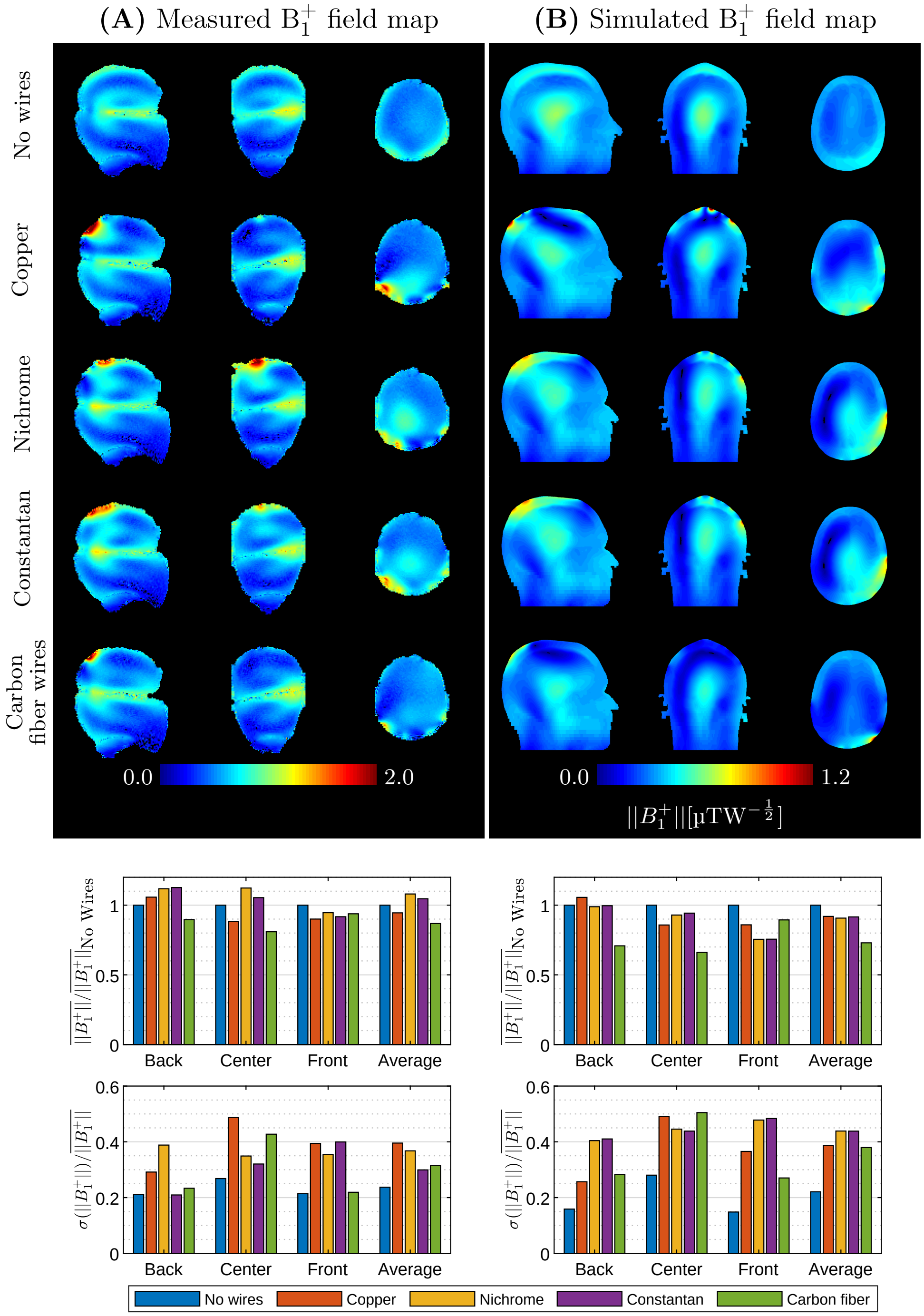

Figure S6: Transmit field maps acquired in the agar-gel phantom, or simulated in the human model with wire-only EEG models. All wiring models produced strong transmit field shielding artifacts compared to no wires, notably at the back of the imaging subject where the wires were bundled. Constantan and nichrome presented a slightly different transmit field disruption pattern related to their thinner insulation layer that affected the propagation and interaction of EM waves, but the attenuation and inhomogeneity were similar to copper. Carbon fiber wires, with the highest linear resistance, presented higher attenuation but a better homogeneity compared to copper.

S7 Voltage across segmentation resistors

The distribution of the peak voltage across segmentation resistors, normalized to 1 W of input power, is plotted in Fig.S7. The maximal peak voltage is 2.35 $VW^{-1/2}$.

On our MRI scanner, the peak power at the coil plug is 5.1 kW (combined 8x1 kW RF amplifiers, with 37% losses between the amplifiers and the coil plug[4]). Neglecting the losses between the plug and coil elements (including the power splitter and TR switches), the maximal voltage across segmentation resistors would be 167 V at full power, which is below the working voltage of these resistors (200 V).

In typical measurements, the coil operates using about 3 kW out of 8 kW combined power at the output of the amplifiers, therefore 1.9kW at the coil plug, resulting in a maximal voltage of 102 V across segmentation resistors.

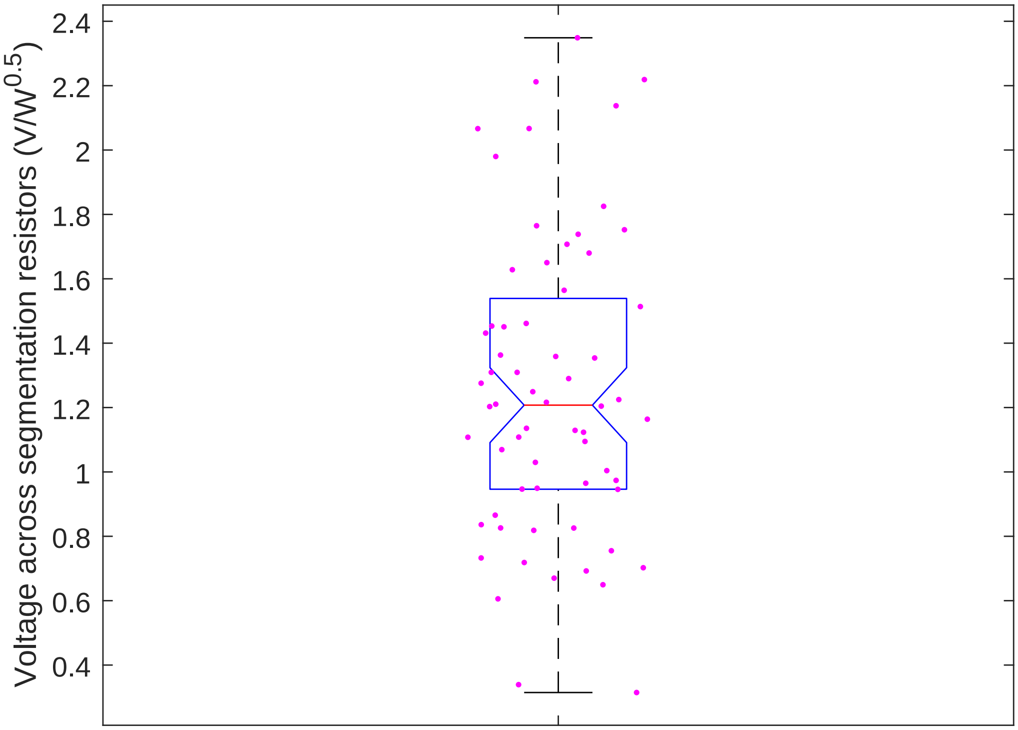

Figure S7: Distribution of the peak voltage across 1 kΩ segmentation resistors, normalized to 1 W of input power. The maximal value, 2.35 $VW^{-1/2}$, corresponds to 167 V at full RF power.

S8 Detailed RF coil models

The RF coil model used in EM simulations, described in SI 1, Fig S1 and Table S1, is a generic 8-loop coil with loops of similar dimensions compared to the physical coil used in MR measurements. The coil model was simplified, in particular each loop was modeled using only three distributed capacitors, and without the concentric shields around each loop. Additional sets of EM simulations using the following two RF coil models were performed to determine whether the simplifications substantially affect the overall $\mathbf{B_1^+}$ field distribution and EEG-induced RF shielding artefacts:

8-loop coil with 14 capacitors per loop: The 8-loop coil was modeled with eight rectangular loops of perfect electric conductors, with an external dimension of $25.0 \times 9.2\text{cm}^2$, and internal size of $23.0 \times 7.2\text{cm}^2$ (Fig. S8a). 14 capacitors were distributed along each loop (Fig.S8b). The length of each PEC segment is about 5cm long, approximately $\lambda/20$. The values of the capacitors and scattering parameters were determined similarly as in S1, and are reported in Fig. S8c and S8d.

8-loop coil with 14 capacitors per loop and concentric shields: The 8-loop coil was modeled with eight rectangular loops of perfect electric conductors of size $24.0 \times 9.0\text{cm}^2$ and 1cm height (Fig. S9a). In contrary to the previous models, the strips of PEC are perpendicular to the patient. 14 capacitors were placed on each loop. A concentric shield modeled as PEC with an overall dimension of $26.0 \times 11.0 \times 2.0\text{cm}^3$ is placed around each loop. The side facing the patient is open, while the opposite face has $24.0 \times 9.0\text{cm}^2$ window. The shield forms a closed loop with a "L" profile placed 1cm away from the loop element (Fig. S9b). All shields are floating (i.e. not grounded, not electrically connected to other concentric shields, any loop elements or any voltage source). The values of the capacitors and scattering parameters are reported in Fig. S9c and S9d.

Simulations with the human model: EM simulations with the above-mentioned coils were performed using the realistic Duke human model as the imaging subject and three EEG configurations (No EEG, Full EEG cap and Segmented full EEG cap). The results are presented in Fig. S10, together with those obtained using the most simple coil model (3 capacitors per loop, described in S1, results from Figs.2 and 7) and MR measurements on the human volunteer for reference (same measurements as on Fig.2). For a given EEG configuration, a similar transmit field distribution was observed together with small local discrepancies, such as with the shielded coil model where the $\mathbf{B_1^+}$ was locally attenuated at the back of the head (arrows 1), and slightly more intense at the front (arrows 2). Nevertheless, with all three coil models, similar EEG-induced RF shielding patterns were observed, with a practically

identical attenuation and inhomogeneity, and similar SAR efficiency (Table S3).

Simulations with the agar-gel phantom : EM simulations with the above-mentioned coils were performed using the digitized agar-gel phantom as the imaging subject and two EEG configurations (No EEG, Full EEG cap). The results are presented in Fig. S11, together with those obtained using the most simple coil model (3 capacitors per loop, results from Fig.3). In overall, a similar transmit field pattern is observed across all three coil models for a given EEG configuration. The full EEG cap cause similar RF shielding patterns with all three coils with slight differences, particularly with the shielded coil for which the $\mathbf{B_1^+}$ at the back of the phantom with EEG is stronger compared to results with the other coil models.

Compared to the more detailed RF coil models, the simplified coil model (SI S1) provided a similar transmit field distribution for a given EEG configuration, and more specifically similar EEG-induced RF shielding patterns. This suggests that the simplified coil model used in this study is a valid approximation of the more detailed RF coil models for the purpose of assessing EEG-induced RF shielding artifacts.

Table S3: SAR efficiency of three coil models simulated with different EEG configurations.

	SAR efficiency ($\mu T kg^{\frac{1}{2}} W^{-\frac{1}{2}}$)		
	No EEG cap	Full EEG cap	Segmented Full EEG cap
3 capacitors per loop	0.570	0.410	0.514
14 capacitors per loop	0.593	0.428	0.527
14 capacitors per loop and concentric shields	0.576	0.427	0.530

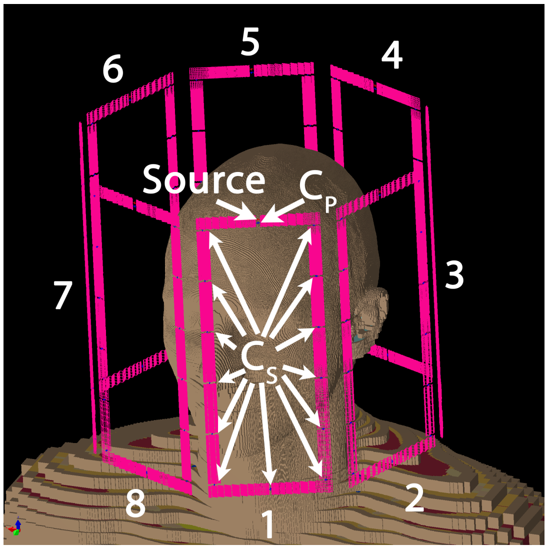

(a)

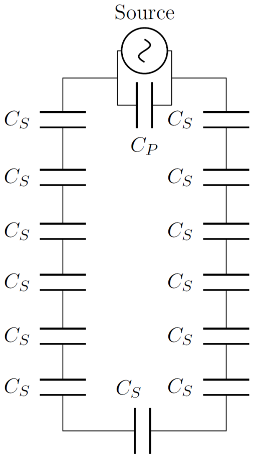

(b)

Loop	Capacitors	
	C_S [pF]	C_P [pF]
1	7.74	10
2	7.75	10
3	7.78	8
4	7.78	4.5
5	7.78	4.5
6	7.78	8
7	7.75	10
8	7.74	10

(c)

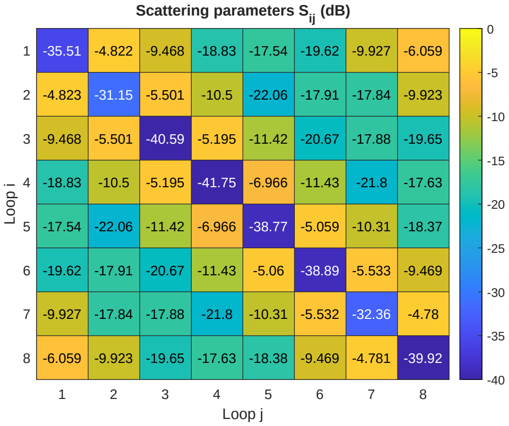

(d)

Figure S8: (a) Voxelized view of the 8-loop RF coil model with 14 capacitors per loop. Numbers indicate the ordering of the voltage sources of the loops. The loops conductive elements are colored in red. (b) Schematic drawing of one of the eight loops of the volume coil array, with the position of the source and 14 capacitors. (c) Values of the tuning and matching capacitors of each loop. (d) Scattering parameters of the simulated coil during transmission.

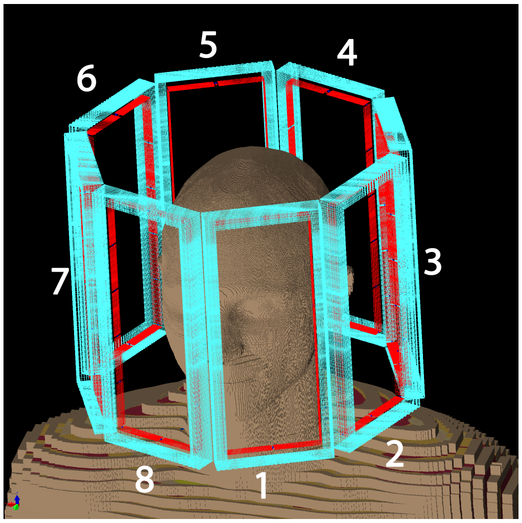

(a)

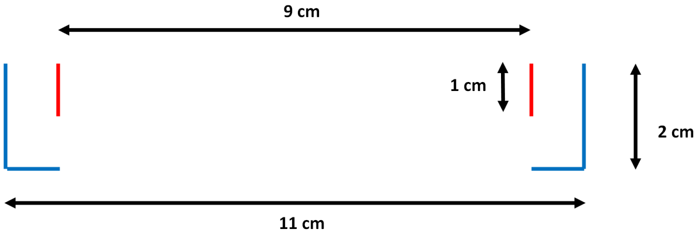

(b)

Loop	Capacitors	
	C_S [pF]	C_P [pF]
1	14.36	23
2	14.36	23
3	14.37	23
4	14.44	21
5	14.44	21
6	14.37	23
7	14.36	23
8	14.36	23

(c)

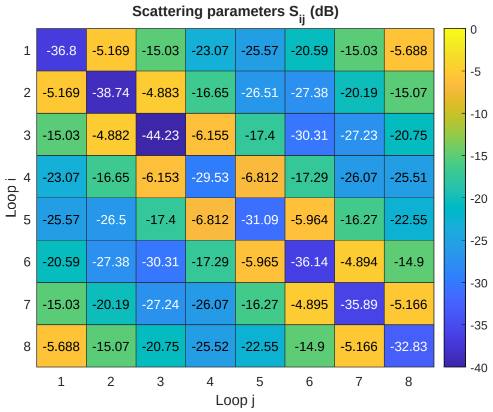

(d)

Figure S9: (a) Voxelized view of the 8-loop RF coil model with 14 capacitors per loop and concentric shields. Numbers indicate the ordering of the voltage sources of the loops. The loops conductive elements are colored in red, while the concentric shields are colored in light blue. (b) Axial cross section of a loop (red) and concentric shield (blue). The spacing between the loop elements and shield is 1cm. (c) Values of the tuning and matching capacitors of each loop. (d) Scattering parameters of the simulated coil during transmission.

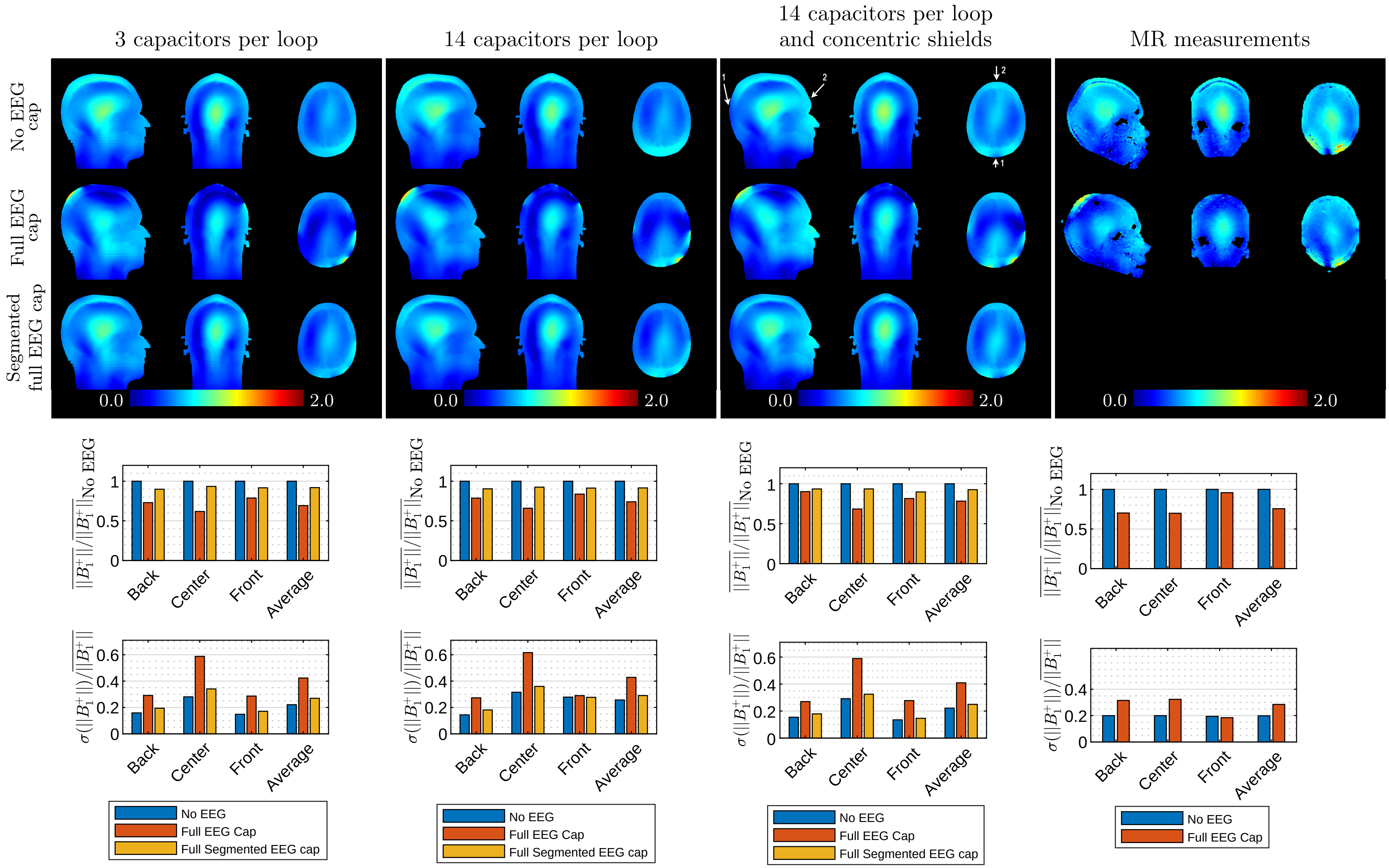

Figure S10: Transmit field maps simulated with three coil models, and different EEG configurations using the realistic human model Duke as imaging subject. MR measurement results from Fig.2 were added for reference on the right-hand side. The transmit field is expressed as a fraction of the nominal flip angle. To enable visual comparison, simulation results were normalized to achieve the same nominal flip angle at the center of the imaging subject in the "No EEG cap" case compared to MR measurements. The same normalization was kept for all simulations with a given coil model. In overall, a similar $\mathbf{B_1^+}$ distribution was observed with all coil models, with local discrepancies, especially with the shielded coil model where a lower and higher $\mathbf{B_1^+}$ are depicted at the back (arrows 1) and front (arrows 2) of the head respectively. Similar RF shielding patterns are observed independently of the coil model.

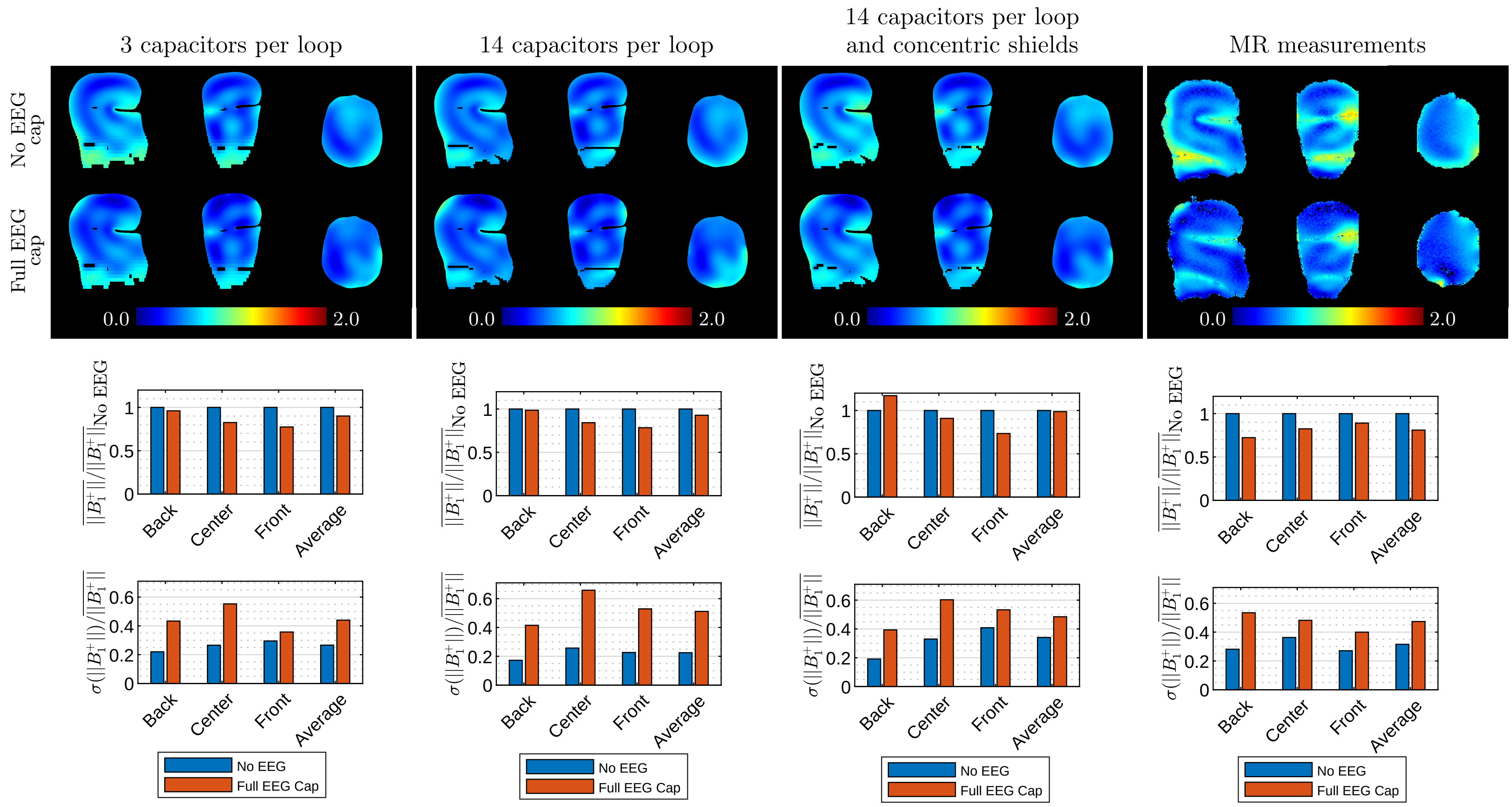

Figure S11: Transmit field maps simulated with three coil models, with and without the full EEG cap, and using the digitized agar-gel phantom as imaging subject. MR measurement results from Fig.3 were added for reference on the right-hand side. All transmit field maps are expressed as a fraction of the nominal flip angle. Simulation results were normalized to achieve the same nominal flip angle at the center of the phantom compared to measurements. In overall, all coil models achieve a similar transmit field distribution with similar RF shielding patterns with all three RF coil models. There are quantitative differences, such as the stronger $\mathbf{B_1^+}$ at the back of the phantom with EEG while using the shielded coil model.

References

[1] Weisser A, and Lanz T. A volume head array with 8 transmit/Receive Channels for 7 T. In Proceedings of the 14th Annual Meeting of ISMRM, 2006. 2591

[2] Kozlov M, Turner R. Fast MRI coil analysis based on 3-D electromagnetic and RF circuit co-simulation. *J Magn Reson.* 2009;200(1):147-52

[3] Jorge J, Grouiller F, Ipek O, et al. Simultaneous EEG-fMRI at ultra-high field: Artifact prevention and safety assessment. *Neuroimage.* 2015;105:132–144.

[4] Clément J, Gruetter R, Ipek Ö. A combined 32-channel receive-loops/8-channel transmit-dipoles coil array for whole-brain MR imaging at 7T. *Magn Reson Med.* 2019;82:1229–1241.
